# Internet addiction, social phobia, substance abuse, and depression in the university setting: a cross-sectional study in the southern region of Morocco

**DOI:** 10.3389/fpsyg.2024.1398989

**Published:** 2024-09-30

**Authors:** Fatima Zahra Ramdani, Laila Lahlou, Mohamed Merzouki, Jalal Doufik, Omar El Oumary, Khadija Akebour, Saliha Hamri, Khalid Mouhadi, Said Boujraf, Hassan Rahioui, Ismail Rammouz

**Affiliations:** ^1^Clinical Neurosciences Innovation and Ethic (NICE), Laboratory REGNE, Faculty of Medicine, Ibn Zohr University, Agadir, Morocco; ^2^Higher Institute of Nursing Professions and Health Techniques, Agadir, Morocco; ^3^Laboratory of Biological Engineering, Department of Life Sciences, Faculty of Science and Technology, Sultan Moulay Slimane University, Beni Mellal, Morocco; ^4^Clinical Neurosciences Laboratory, Faculty of Medicine, Sidi Mohamed Ben Abdellah University, Fez, Morocco; ^5^Department of Neurodevelopmental Disorders, Sainte-Anne University Hospital Pole, Paris, France

**Keywords:** internet addiction, social phobia, substance abuse, depression, prevalence, students

## Abstract

**Aim:**

Internet addiction is a mental health issue that can have detrimental effects on an individual’s life. This study aims to estimate the prevalence of Internet Addiction and identify the risk factors associated with this behavioral addiction.

**Methods:**

This cross-sectional study was conducted in 6 universities in southern Morocco, involving 1,690 students who completed a self-administered questionnaire. The questionnaire collected socio-demographic data, and information on substance use, and included validated tools to assess Internet addiction (Young’s IADQ), depressive symptoms (Patient Health Questionnaire. PHQ-9), and social phobia (Mini Neuropsychiatric International Interview. M.I.N.I).

**Results:**

The findings of our study indicate that the prevalence of Internet addiction was 30.60% (95% CI), depressive symptoms were present in 44.10% of participants, and the prevalence of social Phobia was 30.20%. A significant correlation was identified between early initiation of tobacco and Internet addiction (*p* = 0.05). The multivariate regression model revealed a possible association between cocaine use and Internet Addiction (OR = 5.67, IC 95%: 0.45 to 10.80) (*p* = 0.03), as well as a significant association between social phobia and a higher internet addiction score (OR = 3.45, IC 95%: 1.70–5.02) (*p* < 0.001). However, depressive symptoms were not significantly associated with internet addiction (*p* = 0.38).

**Conclusion:**

These results highlight the urgent need to address internet addiction in the coming years. Implementing Multidisciplinary prevention strategies, early diagnosis, and follow-up measures are essential to mitigate the physical, psychosocial, and academic impacts of this addiction on students.

## Introduction

The growing number of Internet users is indicative of progress toward universal and effective connectivity. As of early 2024, around 5.35 billion people, or over 66.2% of the world’s population, are connected to the Internet (DataReportal – Global Digital Insights, 2024). Today, the Internet plays a crucial role in both personal and professional aspects of life.

However, this widespread connectivity has gradually and unexpectedly become problematic. The intensive use of the Internet reflects a cultural shift in society, where our activities are increasingly dominated by online interactions (Dahl and Bergmark, 2020).

Studies in the literature indicate that the intensive use of the internet is now considered a serious public health issue, as recognized in the fifth edition of the Diagnostic and Statistical Manual of Mental Disorders (DSM-5) and the eleventh revision of the international classification of diseases (IDC-11) (Crocq and Guelfi, 2015; World Health Organization, 2004). This concern arises not only from the consequences faced by individuals but also from the impact on their family members, leading to significant financial difficulties and antisocial behaviors (Calado and Griffiths, 2016). For example, excessive internet use can exacerbate aggressiveness and impulsivity, while internet addiction can develop antisocial behaviors such as isolation and a decrease in social relationships, replacing them with online interactions (Rathi et al., 2022; Chemnad et al., 2023). Additionally, both adolescents and adults may experience physical and psychological health problems related to Internet addiction (Mo et al., 2020; Trumello et al., 2021).

For this study, internet addiction is defined as being addicted to the virtual world of the internet and the inability to control one’s online behavior, which can negatively impact one’s psychology and cause dysfunction in daily life (Lu et al., 2022; Li et al., 2024). Addiction begins when internet use becomes uncontrollable, resulting in a decline in quality of life.

Problematic Internet Use is similar to a psychological dependency, characterized by excessive and uncontrolled usage, whatever the context of use. This behavior can negatively impact an individual’s social integration, with the internet becoming an indispensable tool for adolescents, playing a significant role in their peer integration and identity formation (Khatcherian et al., 2022). Adolescents and young adults are the most frequent users of the Internet (Chung et al., 2019), Making them increasingly vulnerable to excessive use.

However, pathological use of the Internet can lead to various risks, including psychological, social, and emotional harm, as well as negative impacts on academic and professional performance (Chen et al., 2018; Javaeed et al., 2020; Islam et al., 2020). It can also contribute to fatigue, daytime sleepiness due to reduced sleep time, and a diminished perception of quality of life (Ferrari Junior et al., 2024).

Studies conducted in various socio-cultural contexts have shown that problematic internet use can be associated with or exacerbate psychiatric disorders such as depression, social phobia, and substance abuse. It may also intensify symptoms of attention deficit hyperactivity disorder (Xu et al., 2021; Seki et al., 2019; Gupta et al., 2021; Younes et al., 2016; Jaiswal et al., 2020).

A systematic review highlighted that no surveys on gambling behavior have been conducted in many countries (Gabellini et al., 2023). Additionally, Few studies have assessed Internet addiction and its associated factors among university students in Africa (Zewde et al., 2022; Omoyemiju and Popoola, 2021; Arafa et al., 2019; Zenebe et al., 2021).

In this context, a comprehensive analysis report on over 50,000 Asian students from 39 selected studies found that approximately one-fifth suffer from internet addiction. This has been identified as a significant, current, and growing health problem with detrimental effects on students’ physical, emotional, and academic well-being that cannot be ignored (Duc et al., 2024). Similarly, a European literature review on Internet addiction indicates that those most likely to report excessive problematic Internet use are educated adolescents and young men with comorbid disorders (Lopez-Fernandez and Kuss, 2020).

Several studies across different countries have confirmed that, despite socio-cultural differences, internet addiction remains a widespread phenomenon globally.

The 1st meta-analysis examining the epidemiology of Internet Addiction, which included selected studies from 2003 to 2018 across 31 countries, reported an overall prevalence of (7.02%). This prevalence varied over the years, depending on the assessment tools utilized (Pan et al., 2020). In Europe and the United States, prevalence rates range from 1.5 to 8.2% (Weinstein and Lejoyeux, 2010).

On one hand, the online survey conducted by Yang-Yang Li and his team to assess internet addiction in the general population in China during the COVID-19 quarantine revealed an increase in the overall prevalence of internet addiction (36.70%) (Li et al., 2021). On the other hand, a study examining problematic internet use among Japanese adult psychiatric patients indicated that the prevalence of internet addiction in this population is significantly higher, at (25%) compared to just (6%) in the general population (de Vries et al., 2018).

According to the annual report of Morocco’s National Telecommunications Regulatory Agency (ANRT), internet penetration in 2021 was (93.24%) with a target of achieving (100%) coverage by 2024 (Agence Nationale de Réglementation des Télécommunications, 2021). This indicates that Morocco is enhancing its connectivity to keep pace with digitalization and global technological advancements.

A study examining the prevalence of Internet addiction among high-school students in Morocco revealed a rate of 15.80% (Mohamed and Bernouss, 2020). Similarly, among Moroccan medical students, the prevalence of Internet Addiction was reported at 44.5% (Traore et al., 2023). However, studies investigating the prevalence of problematic Internet use and its relationship with associated psychological factors are relatively scarce in Morocco.

The literature shows the existence of some studies investigating the correlation between internet addiction and psychological factors, such as depression, anxiety, social phobia, and substance use. Notably, certain psychological factors, including depression and social phobia, may render young people more vulnerable to internet addiction. A study conducted in Guangdong province elucidates the association between internet addiction and psychological issues, such as depression, anxiety, and stress among adolescents (Xue et al., 2023).

Another study involving college students demonstrates that anxiety predicts internet addiction, which, in turn, predicts depression among male college students (Xie et al., 2023). Additionally, a meta-analysis of 39 studies from various countries suggests that social anxiety is a predictive factor in the development of problematic internet use among adolescents and young adults (Ding et al., 2023).

In this context, the present study aims to estimate the prevalence of internet addiction among university students and to identify the various associated sociodemographic and psychological factors.

## Methods

### Design

This study is an observational, cross-sectional, descriptive, and analytical survey involving a representative sample of 1,690 university students during the 2022–2023 academic year at Ibn Zohr University, a public institution encompassing 05 regions of the Moroccan kingdom. The university is established in 08 towns in southern Morocco through 24 institutions (Université Ibn Zohr, 2024) and includes students from various cities in the Souss-Massa region, such as Agadir, Taroudant, Tiznit, Tata, Ait Baha, as well as cities in the Saharan regions, including Laayoune, Dakhla et Smara.

The survey was conducted at 6 public higher education institutions in the following fields: Faculty of Medicine and Pharmacy, Faculty of Arts and Humanities, Faculty of Sciences, National School of Engineering, Faculty of Law, Economic and Social Sciences, and Institute of Nursing.

Students in the 1st, 2nd, and 3rd cycles were eligible to participate in the survey, provided they were Moroccan nationals and regularly enrolled in one of the selected institutions. However, Foreign students were excluded from the study.

Before collecting the data, we reviewed the scheduling of theoretical courses and practical work to ensure that the survey periods did not coincide with exam dates. Subsequently, we visited the classrooms to assess the arrangement of the tables, ensuring that on the day of the survey, students would be seated at a sufficient distance to minimize any bias that could affect the confidentiality of their responses.

### Target population

The target population comprises all students at IBN ZOHR University (UIZ), which represents 50% of the total university student population in Morocco. The university has approximately 151,000 students, from which our sample was selected.

### Sampling method

We conducted a random draw from all the university establishments affiliated with Ibn Zohr University in the Agadir region, as this city is the most accessible for our team and houses the largest number of training establishments (a total of 10).

From this selection, we randomly chose 06 public institutions from all the public establishments in Agadir to recruit our sample, utilizing lists provided by the administration of each selected university.

On the official day of the survey, students who were present in the lecture halls and classrooms and who had scheduled classes on that day were recruited to participate in our survey.

### Sample size

To calculate the sample size, we used an alpha risk of 5%, a precision of 0.03, and a prevalence of Internet Addiction estimated in the literature at 40.33% (Endomba et al., 2022). We calculated a minimum sample size of 1,028 (Cochran and Cochran, 1977; Bartlett et al., 2001), adding 39% to this minimum to arrive at a final size of 1960 participants to avoid the problem of missing data.

### Concerning students refusing to participate in the study

The response rate was 100%, with no students refusing to complete the questionnaire. However, some questions had missing data due to certain participants choosing not to respond.

### Measures

A self-administered socio-demographic questionnaire, written in French, was used in this study. The questionnaire was paper. With an estimated average completion time of 15 min, as determined by a pre-test. It consists of a series of valid and reliable tests, organized into 4 sections, the 1st section was inspired by the questionnaire from the Moroccan survey Mediterranean School Project on Alcohol and Other Drugs (MedSpad) (El Omari et al., 2022).

The Questionnaire items include socio-demographic information (age, gender, university, place of residence, grade repetition, class absenteeism, parental socio-economic status). This is followed by questions addressing lifetime use of substances such as tobacco, hookah, sucking tobacco, alcohol, cannabis, benzodiazepines without medical prescription, cocaine, and heroin.

In part 2, we used the Internet Addiction Diagnostic Questionnaire (IADQ), developed by Young (1998), to identify abnormal internet use. The IADQ comprises 8 dependency criteria, each answered with a Yes or No. A total score of more than 5 (Yes) responses indicates internet addiction.

The 8 items of this tool addressed the following aspects: (preoccupation with the Internet, the need to surf the Internet for long periods; Failure to limit control or stop Internet use; Feeling tired, anxious, and depressed when limiting or stopping Internet use; Staying connected longer than originally planned; Putting personal relationships, profession and opportunities at risk because of Internet use; Hiding Internet addiction from family, friends, and caregivers; Using the Internet to escape from life problems or negative feelings).

In part 3, we utilized the Patient Health Questionnaire (Kroenke et al., 2001) to screen for depressive symptoms. This self-administered version consists of 9 items based on the fourth edition of the Diagnostic and Statistical Manual of Mental Disorders (DSM-IV).

Each Item is scored on a scale from 0 to 3 (0 = not at all; 1 = a few days; 2 = more than half the days; 3 = almost every day), resulting in a total score ranging from 0 to 27. Participants are asked to rate the severity of their symptoms experienced over the past 2 weeks. While, the results do not constitute a confirmed medical diagnosis, but a screening of symptom levels, a total score of 10 points or higher indicates the presence of symptoms of severe depression.

Finally, for part 4, we utilized the Mini International Neuropsychiatric Interview (M.I.N.I) based on the DSM-IV to assess the presence of substance abuse or dependence, as well as social phobia (Kadri et al., 2005). We organized survey teams primarily composed of medical students, each supervised by two psychiatric resident doctors who provided clear instructions to participants on how to answer the M.I.N.I regarding social phobia. To evaluate social phobia, we used the 4 yes/no criteria of the M.I.N.I. A participant was considered to have a current social phobia if they answered yes to the 4th criterion (Does this fear disrupt your normal work or social functioning or cause you significant distress?).

### Ethical considerations

The research protocol for this study adheres to the stringent ethical standards outlined in the Helsinki Declaration and has been approved by the Ethics Committee for Biomedical Research at the Faculty of Medicine and Pharmacy of Rabat, Morocco, under reference No. 51–23. Before starting the study, we obtained an agreement from the presidency of Ibn Zohr University to conduct surveys at the universities in the Agadir region of Morocco.

To minimize information bias, a single interviewer trained to collect data uniformly, conducted all interviews. Participation in the survey was completed voluntarily and unpaid, adhering to ethical standards. Anonymity and confidentiality were strictly maintained and there was no intervention with participants, no physical danger, or disclosure of privacy. Each participant received a briefing note explaining the study’s context, purpose, interest, and ethical considerations. Informed consent was obtained through a signed written consent from each participant.

### Statistical analysis

Data entry and analysis were performed using Jamovi 2.4.11 software, which operates on the R programming language. The software includes built-in automatic adjustments for alpha correction, eliminating the need for manual peer-to-peer testing or additional alpha corrections, as these are handled automatically.

Qualitative variables were described using frequencies and percentages and were compared using the chi-square test or Fisher’s exact test, depending on the conditions of application of each test.

Quantitative variables following a Gaussian distribution were described as mean ± standard deviation and compared using Student’s t-test. The significance level was set at 5%. The distribution of quantitative variables was assessed based on graphical representations (histogram and QQ Plot). Prevalence rates, along with their 95% confidence interval, were estimated using the esci module in Jamovi software.

Finally, to determine the factors associated with variations in the increase in the Internet addiction score, we conducted both univariate and multivariate linear regression analyses. Factors that had a *p*-value of ≤0.05 were included in the multivariate model. Given the exploratory nature of our study, we performed numerous univariate analyses on a variable-by-variable basis without applying correction for multiple comparisons. The aim was to identify a wide range of variables likely to be associated with internet addiction. Future studies will be needed to confirm these associations.

## Results

A total of 1,690 students from various universities in the Agadir region of Morocco participated in our survey. Approximately 1,099 students (65.0%) were female. The average age of participants was 20.3 ± 3.21 years.

Table 1 provides a detailed description of the socio-demographic and clinical characteristics of the participants. Of these, (56.6%) were still living with their parents, (83.3%) had never repeated a year at university, and (77.5%) of the students perceived themselves as coming from families with an average socio-economic status.

**Table 1:** Descriptive data for university students participating in the study (*n* = 1,690).

Variables	*Number (%)
Age in years	20.3 ± 3.21^Δ^
Gender	
Female	1,099 (65.0)*
Male	591 (35.0)*
Institutions	
Medicine	327 (19.3)*
Humanities	382 (22.6)*
Sciences	253 (15.0)*
Nursing institute	431 (25.5)*
Law sciences	260 (15.4)*
Residency	
Boarding	157 (09.3)*
With parents	956 (56.6)*
With friends	577 (34.1)*
Repeat class	
Yes	283 (16.7)*
No	1,407 (83.3)*
Socioeconomic level	
Higher	208 (12.3)*
Medium	1,310 (77.5)*
Lower	172 (10.2)*
Tobacco	
Lifetime use	158 (09.4)*
Hookah	
Lifetime use	110 (6.5)*
Chewing tobacco	
Lifetime use	37 (02.2)*
Inhaling tobacco	
Lifetime use	33 (02.0)*
Alcohol	
Lifetime use	77 (04.6)*
Cannabis	
Lifetime use	29 (01.7)*
Benzodiazepine	
Lifetime use	53 (03.1)*
Cocaine	
Lifetime use	05 (0.3)*
Heroine	
Lifetime use	02 (0.1)*
Depressive symptoms	
No	929 (55.9)*
Yes	732 (44.1)*
Social phobia	
No	1,164 (69.8)*
Yes	504 (30.2)*
Internet addiction	
No	1,159 (69.4)*
Yes	510 (30.6)*

^ΔMean ± Standard deviation.^

*Indicates the percent (%).

In our study, tobacco has the highest lifetime use prevalence among students at (9.4%) 95 CI [8.0–10.8%], with an average age of 1st cigarette use at 16.6 years ±2.98. Followed by hookah with a prevalence of (6.5%) 95 CI [5.4–7.8%] and cannabis at (1.7%) 95 CI [1.1–2.4%]. Notably, (75.8%) of cannabis users reported preferring to consume it in the form of joints (Table 1).

Table 2 presents the descriptive data and internal consistency measures of the internet addiction score, which demonstrated a high level of internal consistency (Cronbach’s *α* = 0.81).

**Table 2:** Descriptive data and internal consistency of the internet addiction score for university students participating in the study (*n* = 1,690).

Variable	Mean	Median	SD	Variance	Internal consistency (Cronbach’s α)
Internet addiction score	3.75	4	2.62	6.87	0.81

The prevalence of internet addiction among students from the participating universities was (30.60%) 95 CI [28.39–32.81%], followed by the prevalence of symptoms of severe depression was (44.10%) 95 CI [41.70–46.50%] and the prevalence of social phobia was (30.20%) 95 CI [28.06–32.50%].

The association between social phobia and internet addiction was significant (*p* < 0.001), with (51.4%) of students with social phobia classified as internet addicts (Table 3).

**Table 3:** The association between internet addiction, depression, and social phobia (*n* = 1,690).

	Internet addiction (+)		Internet addiction (−)		Statistical test	Degree of freedom	*P*-value
Depressive symptoms	Present	228 (45.7)*	Present	495 (43.4)*	Chi-squared test	1.00	0.38
	Absent	271 (54.3)*	Absent	646 (56.6)*			
Social phobia	Present	261 (51.4)*	Present	243 (21.0)*	Chi-squared test	1.00	<0.001
	Absent	247 (48.6)*	Absent	912 (79.0)*			

*Number (%).

In contrast, the association between depressive symptoms and Internet Addiction was not significant among our participants (*p* = 0.38) (Table 3), despite (44.1%) of the students having symptoms of depressive disorders (Table 1).

There was also a significant association between younger age of first use of inhaled tobacco and internet addiction among students (*p* = 0.05) (Table 4).

**Table 4:** Comparison analysis of internet addiction among students (*n* = 1,690).

	Internet addiction (+)		Internet addiction (−)		Statistical test	Degree of freedom	*P*-value
Age in (years)	20.2 ± 2.53^Δ^		20.4 ± 3.42^Δ^		Welch *t*-test	1292.07	0.19
Sex	F	343 (67.3)*	F	749 (64.6)*	Chi-squared test	1.00	0.29
	M	167 (32.7)*	M	410 (35.4)*			
Initiation age cigarette	16.0 ± 2.51^Δ^		16.8 ± 3.15^Δ^		Student’s *t*-test	154.00	0.13
Tobacco	Yes	49 (9.6)*	Yes	108 (9.3)*	Chi-squared test	1.00	0.86
	No	461 (90.4)*	No	1,049 (90.7)*			
Initiation age Hookah	15.8 ± 4.23^Δ^		16.9 ± 3.35^Δ^		Student’s *t*-test	109.00	0.15
Hookah	Yes	37 (7.3)*	Yes	73 (6.3)*	Chi-squared test	1.00	0.47
	No	473 (92.7)*	No	1,084 (93.7)*			
Initiation age Chewing Tobacco	16.4 ± 2.51^Δ^		17.2 ± 3.46^Δ^		Student’s *t*-test	35.00	0.50
Chewing Tobacco	Yes	9 (1.8)*	Yes	28 (2.4)*	Chi-squared test	1.00	0.40
	No	501 (98.2)*	No	1,129 (97.6)*			
Initiation age Inhaling Tobacco	15.3 ± 3.11^Δ^		18.0 ± 3.19^Δ^		Student’s *t*-test	27.00	0.05
Inhaling Tobacco	Yes	10 (2.0)*	Yes	23 (2.0)*	Chi-squared test	1.00	0.97
	No	500 (98.0)*	No	1,134 (98.0)*			
Initiation age alcohol	18.2 ± 1.42^Δ^		18.9 ± 2.87^Δ^		Welch *t*-test	28.71	0.17
Alcohol	Yes	18 (3.5)*	Yes	59 (5.1)*	Chi-squared test	1.00	0.15
	No	492 (96.5)*	No	1,098 (94.9)*			
Initiation age cannabis	16.9 ± 1.60^Δ^		18.2 ± 3.89^Δ^		Student’s *t*-test	61.00	0.14
Cannabis	Yes	11 (2.2)*	Yes	18 (1.6)*	Chi-squared test	1.00	0.38
	No	499 (97.8)*	No	1,139 (98.4)*			
Initiation age Benzodiazepine	17.8 ± 2.88^Δ^		17.4 ± 4.60^Δ^		Student’s *t*-test	51.00	0.70
Benzodiazepine	Yes	22 (4.3)*	Yes	31 (2.7)*	Chi-squared test	1.00	0.08
	No	488 (95.7)*	No	1,126 (97.3)*			
Initiation age Cocaine	17.0 ± 3.46^Δ^		17.0 ± 1.41^Δ^		Student’s *t*-test	3.00	1.00
Cocaine	Yes	3 (0.6)*#	Yes	2 (0.2)*	Fisher’s exact test	1.00	0.17
	No	507 (99.4)*	No	1,155 (99.8)*			
Heroine	Yes	0 (0.0)*	Yes	2 (0.2)*	Fisher’s exact test	1.00	1.00
	No	510 (100.0)*	No	(99.8)*			

*Number (%) ^Δ^[Mean ± Standard deviation] (+) Present (−) Absent. #Due to the low number of participants reporting cocaine use and Internet addiction (*n* = 3). Fisher’s exact test was used for the analysis, and the results should be interpreted with caution, as the small number of cases limits the generalizability of the findings.

Similarly, both univariate and multivariate regression models applied to the Internet addiction test score described that, in our university study, cocaine use (*p* = 0.03) and social phobia (p < 0.001) were factors associated with an increase in the Internet addiction score (Table 5). Conversely, no positive correlation was found between internet addiction and the use of tobacco, chewing tobacco, inhaling tobacco, hookah, alcohol, cannabis, benzodiazepines without prescription, and heroin.

**Table 5:** Factors associated with variation in internet addiction scores among students (*n* = 1,690).

Variables		Beta	95% IC	*p*-value	Beta (adjusted)	95% IC	*p*-value
Age (in years)		−0.04	−0.083; −0.004	0.03	−0.13	−0.31; 0.05	0.16
Sex M-F		−0.20	−0.46; 0.064	0.13			
Faculty	Arts-medicine	0.4	0.01; 0.78	0.04	−0.85	−4.15; 2.43	0.60
	Sciences-medicine	0.18	−0.24; 0.61	0.40	−0.066	−3.03; 2.89	0.96
	Engineering-medicine	−0.90	−1.80; −0.01	0.04	−0.97	−4.31; 2.36	0.55
	Nursing-medicine	0.91	0.54; 1.28	<0.001	−0.76	−4.23; 2.71	0.66
	Law and economics-medicine	−0.43	−0.85; −0.006	0.047	−1.63	−4.80; 1.52	0.30
Half year	2–1	0.76	0.32; 1.20	<0.001	−1.43	−4.99; 2.11	0.41
	3–1	−0.75	−1.40; −0.09	0.03	0.10	−2.83; 3.03	0.94
	4–1	0.10	0.67; 1.32	<0.001	−0.63	−3.19; 1.92	0.62
	5–1	0.70	0.15; 1.25	0.013	−0.68	−3.30; 1.93	0.60
	6–1	0.81	0.41; 1.22	<0.001	−1.51	−4.86; 1.83	0.36
	9–1	−0.20	−0.81; 0.42	0.53			
Socioeconomic level	Medium—Higher	0.60	0.21; 1.0	0.002	−0.70	−2.86; 1.44	0.51
	Lower—Higher	1.32	0.80; 1.86	<0.001	−1.82	−4.52; 0.87	0.18
Repeater		0.20	−0.14; 0.53	0.25			
Initiation first cigarette		−0.15	−0.30; −0.01	0.037	−0.10	−0.43; 0.22	0.52
Smoking last 12 months		0.28	−0.27; 0.83	0.31			
Cocaine use		2.05	−0.25; 4.35	0.08	5.67	0.45; 10.80	0.03
Benzodiazepine use		0.33	−0.40; 1.05	0.37			
Initiation age alcohol		−0.27	−0.50; −0.04	0.02	0.24	−0.22; 0.70	0.29
Friend use alcohol		0.81	0.52; 1.10	<0.001	1.27	−1.23; 3.78	0.31
Family use alcohol		0.65	0.28; 1.03	<0.001	1.08	−0.64; 2.80	0.21
Social phobia		1.82	1.56; 2.08	<0.001	3.45	1.70; 5.02	<0.001
Depressive symptoms		0.10	−0.15; 0.35	0.45			

Confidence Interval = 95%.

## Discussion

This study found that the prevalence of Internet Addiction in Morocco is 30.6% as measured by Young’s Internet Addiction Diagnostic Test. A Moroccan study conducted among students at the Casablanca Faculty of Medicine reported a higher prevalence of Internet Addiction (44.5%) (Traore et al., 2023) compared to our finding in Agadir. In contrast, another similar survey among Moroccan high school students revealed a lower prevalence rate of (15.80%) (Mohamed and Bernouss, 2020) compared to our student sample.

In a survey of 408 students at a Korean university, the prevalence rate of internet addiction was (11.5%) (Seo et al., 2021). At a university in Nepal, the prevalence was (29%) among a sample of 344 students (Adhikari et al., 2022). In Pakistan, the prevalence was (7.9%) among 148 students (Haroon et al., 2018).

The prevalence of Internet Addiction among students varies across countries, different schools, and institutions, ranging from (0.8% à 34%) (Cash et al., 2012).

Despite the absence of common criteria for screening Internet Addiction and the diversity of measurement tools, numerous epidemiological studies have been conducted to assess the extent of excessive Internet use.

In the literature, we found 22 studies from 7 countries (Egypt, Ethiopia; Nigeria; South Africa; Tunisia; Morocco) (Endomba et al., 2022) that investigated Internet addiction. The prevalence of Internet addiction varied from 12.33 to 87.67%, with sample sizes ranging from 120 to 1,600 participants. The results are not completely comparable with ours due to the variation in the tools used to measure internet addiction. However, Tunisian studies; which applied a similar methodology and the same data collection tools as our study, reported a variation in prevalence ranging from 18.06 to 43.87% (Boudabous et al., 2020; Ben Thabet et al., 2019; Chérif et al., 2015; Ellouze et al., 2015). This underscores the importance of contextualizing internet addiction rates according to the specific characteristics of each population.

Our study analyzed factors associated with Internet Addiction, including social phobia, cocaine use, and early onset of smoking. Among the participants, 732 (44.1%) announced symptoms of severe depression, and 228 (45.7%) of these were students with both depressive symptoms and Internet Addiction. However, the analysis of the association between depressive symptoms and internet addiction in our study was non-significant (*p* = 0.38).

Carly et al. conducted a systematic review to identify and evaluate studies performed on the correlation between Internet addiction and comorbid psychopathology. They reported twenty articles, finding significant correlations in 75% of studies with depression, 57% with anxiety, 100% with symptoms of Attention Deficit Hyperactivity Disorder (ADHD), 60% with obsessive-compulsive symptoms, and 66% with hostility/aggression (Carli et al., 2012).

Similarly, studies conducted among university students have demonstrated a significant association between internet addiction and depression, such as the study conducted among students at the Oman Medical Specialty Board (Al Mukhaini et al., 2021).

Another study analyzed the link between high scoring of internet addiction and its impact on the depression treatment process, revealing that students with severe depression accompanied by Internet Addiction will have less effective therapeutic outcomes compared to those with mild depression. In other words, internet addiction and depression both affect the duration and efficacy of treatment (Zhou et al., 2023).

Likewise, a large-scale survey conducted in Chengdu among Chinese adolescents indicated a high probability of developing depression among those with Internet addiction (Zhao et al., 2023), identifying this addiction as a risk factor for subsequent depression.

Comparing our results with other studies carried out in the same country, Morocco, we note that very few have explored the link between internet addiction and psychological factors. One such study, a cross-sectional analysis conducted at the Faculty of Medicine and Pharmacy in Casablanca (Traore et al., 2023), found a depression rate of (44.5%) among students, which is similar to the rate close to found in our ample (44.1%).

On another note, the link between internet addiction and substance abuse remains a topic of debate, as they share a common set of addiction criteria. Could it be a substitution of one addiction for another, or a continuum of a pattern of use through the ages, or is it a particular psychopathology of a behavioral addiction that differs from substance dependence? Studies have established this link (Semaille, 2009). Drug use may predict a high risk for internet addiction, as students with at risk for internet addiction often present a vulnerability to addictive behaviors. Consequently, co-morbid substance abuse should be evaluated and treated in adolescents with internet addiction (Lee et al., 2013). In another study, Lee and Lee confirmed significant associations between addictive use of the Internet at the age of 15 and heavy drinking and cigarette smoking at age 20, highlighting the negative effects of addictive Internet use as one of the major problems facing adolescents (Lee and Lee, 2017).

However, in our survey, the analysis of the relationship between substance use and internet addiction revealed no significant associations, except for the association between internet addiction and the age of 1st inhaled tobacco use (*p* = 0.05).

In multivariate analysis, a possible association was found between cocaine use and Internet dependence (OR = 5.67, IC95%:0.45–10.80) (*p* = 0.03). Few studies have explored the link between internet addiction and the increased risk of cocaine use. Among the most notable is a large national cohort study of a large sample of Mercian students, which analyzed the relationship between problematic internet use and substance abuse, finding that students reporting internet addiction had an elevated risk of cocaine abuse (Qeadan et al., 2022).

Similarly, our multivariate regression analysis proved a high-risk association between social phobia and increased internet dependence score (OR = 3.45, IC95%: 1.70–5.02) (*p* < 0.001). Notably, (30.2%) of participants presented social phobia, with (51.4%) of those identified as internet-dependent students. Other studies have confirmed the link between social phobia and internet addiction among adolescents and young adults (Yen et al., 2007).

A study carried out on a large scale among online games who interact by creating their avatar, demonstrated that social phobia among these gamers contributes to internet gambling disorders. The creation of a fictional avatar serves as a manifestation of the gamer’s ideal self, satisfying their need for social interaction while managing social anxiety (Sioni et al., 2017). Therefore, young people suffering from social phobia may invest in interpersonal relationships yet often remain socially isolated, making them more susceptible to internet addiction as a solitary and pleasurable activity. In the same way, when internet users turn to the internet as a means of coping with loneliness, they may find it easier to lose control over their internet use (Hernández et al., 2024).

Consistent with our findings, a study involving a diverse group of students in India found a correlation between social phobia and internet dependence among those identified as internet-dependent (Jaiswal et al., 2020). additionally, a survey conducted on a population of online gamers revealed that those spending excessive hours online exhibited high scores for both social phobia and internet dependence (Wei et al., 2012). Similarly, a study conducted during the COVID-19 pandemic among adolescents and young online gamers in several Arab countries revealed a strong association between online gaming addiction and elevated social phobia scores resulting from isolation (Shaheen et al., 2023).

## Strengths and limitation

Although this study provides significant results regarding the prevalence of internet addiction among students in southern Morocco with a large sample size, it also has both limitations and strengths.

Firstly, the study is limited by its cross-sectional design, which offers a single point of assessment, making it difficult to draw definitive conclusions regarding the links among drug use, social phobia, depression, and problematic internet use. This limitation highlights the need for further longitudinal research. Secondly, the method of recruiting students relied on the completion of a self-administered questionnaire, which may be susceptible to recall bias.

Another limitation is the absence of a diagnostic interview and the lack of objective measures. This study represents a preliminary step in measuring problematic internet use among Moroccan students and is one of the few studies to examine the relationship between internet addiction and psychological factors.

These limitations suggest that future longitudinal studies will be necessary.

## Conclusion

The prevalence of internet addiction among students in our study was found to be high, with a possible association observed with cocaine use, social phobia, and early tobacco use. This finding prompted health authorities to intensify their efforts in preventing the development of various forms of internet addiction to guarantee the physical and psychological well-being of young people. Effective multidisciplinary management of internet addiction could serve as a butterfly effect, leading to positive outcomes in the physical, psychological, and social well-being of young students.

## Data Availability

The raw data supporting the conclusions of this article will be made available by the authors, without undue reservation.
